# Proteomic Interactions in the Mouse Vitreous-Retina Complex

**DOI:** 10.1371/journal.pone.0082140

**Published:** 2013-11-29

**Authors:** Jessica M. Skeie, Vinit B. Mahajan

**Affiliations:** 1 Omics Laboratory, University of Iowa, Iowa City, Iowa, United States of America; 2 Department of Ophthalmology and Visual Sciences, University of Iowa, Iowa City, Iowa, United States of America; UAE University, Faculty of Medicine & Health Sciences, United Arab Emirates

## Abstract

**Purpose:**

Human vitreoretinal diseases are due to presumed abnormal mechanical interactions between the vitreous and retina, and translational models are limited. This study determined whether nonstructural proteins and potential retinal biomarkers were expressed by the normal mouse vitreous and retina.

**Methods:**

Vitreous and retina samples from mice were collected by evisceration and analyzed by liquid chromatography-tandem mass spectrometry. Identified proteins were further analyzed for differential expression and functional interactions using bioinformatic software.

**Results:**

We identified 1,680 unique proteins in the retina and 675 unique proteins in the vitreous. Unbiased clustering identified protein pathways that distinguish retina from vitreous including oxidative phosphorylation and neurofilament cytoskeletal remodeling, whereas the vitreous expressed oxidative stress and innate immunology pathways. Some intracellular protein pathways were found in both retina and vitreous, such as glycolysis and gluconeogenesis and neuronal signaling, suggesting proteins might be shuttled between the retina and vitreous. We also identified human disease biomarkers represented in the mouse vitreous and retina, including carbonic anhydrase-2 and 3, crystallins, macrophage inhibitory factor, glutathione peroxidase, peroxiredoxins, S100 precursors, and von Willebrand factor.

**Conclusions:**

Our analysis suggests the vitreous expresses nonstructural proteins that functionally interact with the retina to manage oxidative stress, immune reactions, and intracellular proteins may be exchanged between the retina and vitreous. This novel proteomic dataset can be used for investigating human vitreoretinopathies in mouse models. Validation of vitreoretinal biomarkers for human ocular diseases will provide a critical tool for diagnostics and an avenue for therapeutics.

## Introduction

Vitreoretinal diseases encompass blinding conditions due to abnormal interactions between the inner surface of the neurosensory retina and the overlying vitreous gel. Abnormalities of the vitreous-retina complex that cause vision loss include posterior vitreous detachment, retinal tear, retinal detachment, vitreomacular traction, macular hole, idiopathic and secondary epiretinal membrane, proliferative vitreoretinopathy, and proliferative diabetic retinopathy. Vitreous-retina interactions also modulate diabetic macular edema and age-related macular degeneration[1,2]. The abnormal mechanical traction of the vitreous on the retina is presumed to be the underlying factor in these diseases. Surgical therapy is the standard interventional approach, but molecular therapy has the potential to improve visual outcomes and overcome limitations of surgery. 

The vitreous gel is an extracellular matrix whose function after development is not known. It is conceivable that, like other extracellular matrices, the vitreous has important biological funcitons. Vitreous proteins may originate from the retina, ciliary body, lens, retinal pigmented epithelium, or the systemic circulation[3,4]. In vitreoretinal disease, the gel composition changes and some proteins are differentially expressed[5-7]. Vitreous biopsies are collected during surgery and used for diagnostic tests for cancer, infection, and autoimmunity. An ELISA of specific vitreous proteins could be used for disease diagnostics. Serum ELISA assays have been developed as cancer diagnostic tests, such as those for MUC5AC and NPC-1C to identify tumor growth in colorectal and pancreatic cancers or C-erbB-2 in breast cancer[8,9]. Retinal proteins, such as vascular endothelial growth factor (VEGF), can be detected in the vitreous of angiogenic vitreoretinopathies using an ELISA assay. The success of anti-VEGF therapy in vein occlusions and diabetic retinopathy supports the search for biomarkers in other vitreoretinal diseases. 

While ELISA assays focus on a single, predetermined molecule, proteomic assays screen thousands of proteins. Using tandem mass spectrometry, proteomic analyses are applied to vitreous samples collected at the time of vitrectomy surgery. Recent studies suggest needle biopsy samples are sufficient for proteomics[10]. By comparing vitreous samples from different diseases, it is possible to identify novel proteins with diagnostic or therapeutic potential. Protein expression patterns may also give critical insight to disease processes. 

**Figure 1:**
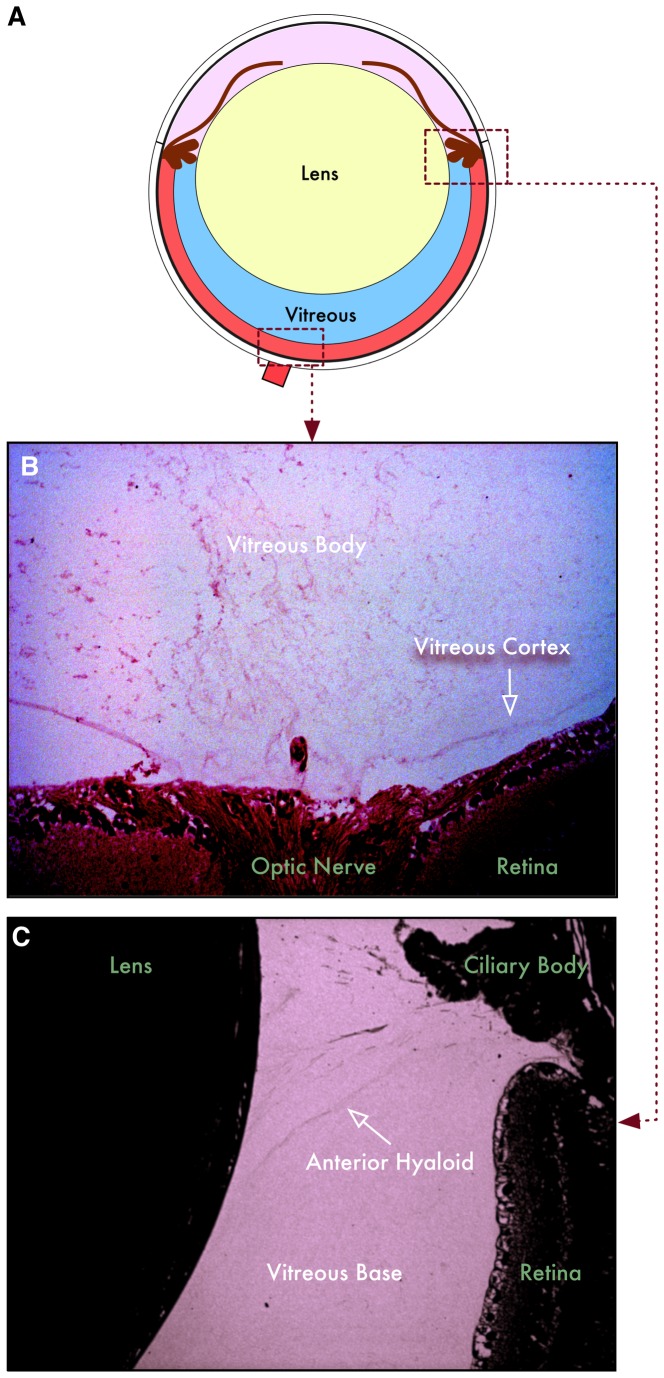
Structure of mouse vitreous. **A**. Illustration of the mouse eyeball. The lens composes a large portion of the eye, leaving a small portion of the eye to be filled with vitreous. **B**. The posterior mouse vitreous has a cortex and body similar to the human vitreous. The cortex defines the vitreoretinal boundary in both human and mouse. **C**. The mouse anterior hyaloid lies between the ciliary body and lens, posterior to the zonules, anterior to the vitreous base.

Human vitreous proteomics has rapidly expanded the list of potential protein biomarkers and molecular disease pathways. However, validation of these biomarkers remains a significant challenge. Human surgical samples are very limited, so other biological models are needed. Molecular genetic manipulations of the mouse have made it an important model for ocular disease, but the mouse has not been frequently used to study vitreous-retinal disease [11]. First, the relative vitreous volume is significantly smaller in mouse eyes when compared to human eyes (Figure 1). Second, its molecular composition is unknown. We previously developed a technique for the isolation of the mouse vitreous sufficient for proteomic analyses[3]. For this study, we used a proteomic approach to reveal the vitreous-retinal proteome of the mouse. The purpose of this study was to develop a dataset of the mouse vitreous and retinal proteomes for future validation of human vitreoretinal disease biomarkers in mouse models.  A similar strategy to compare the proteomes of cells and their adjacent extracellular matrix could be applied to other tissues, such as joints where the fluid filled cavities can be biopsied for biomarker identification.

## Methods

All experiments were performed in accordance with the ARVO Statement for the Use of Animals in Ophthalmic and Visual Research and were all approved by the Animal Care and Use Committee at the University of Iowa.

### Mouse vitreous and retina sample collection

**Figure 2:**
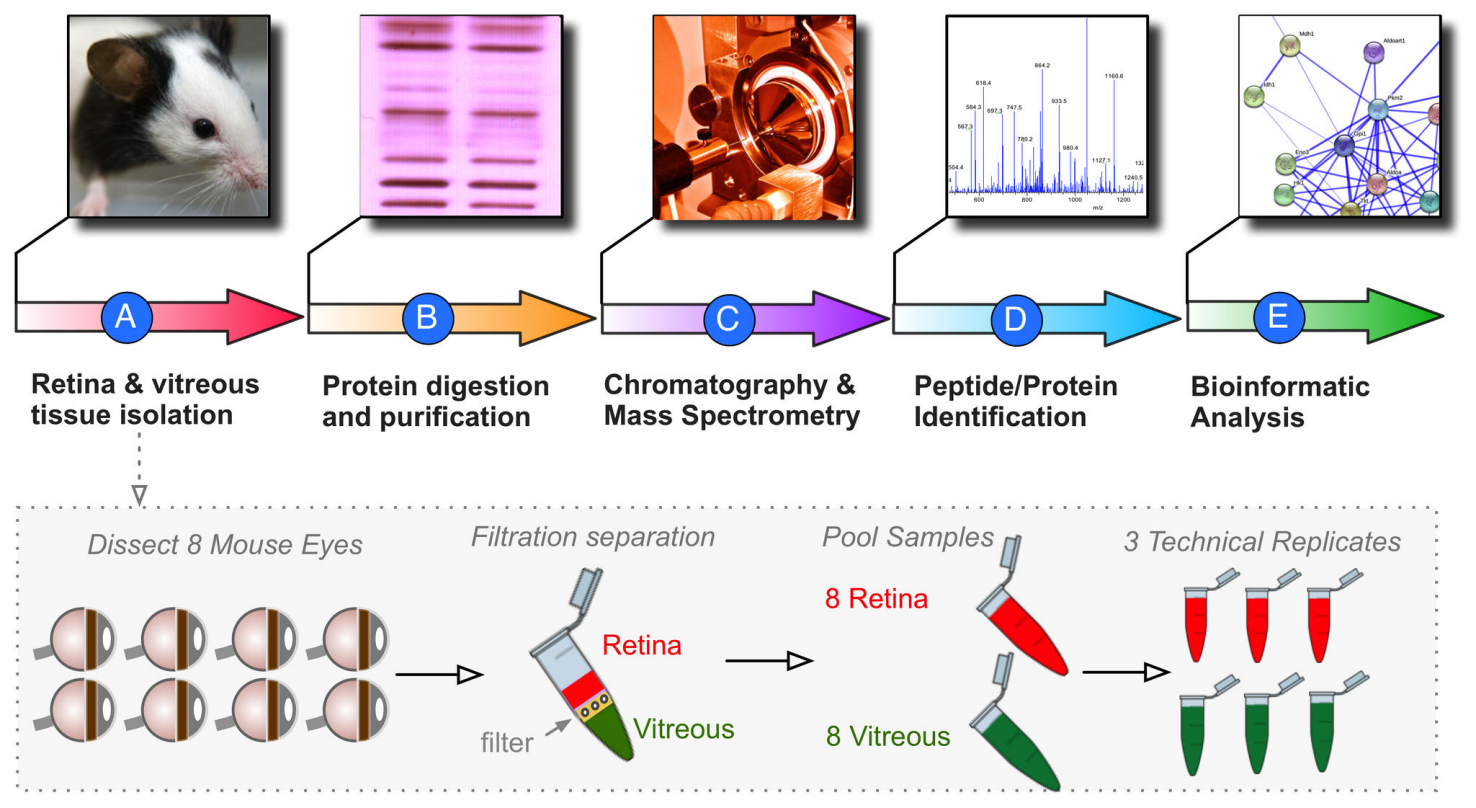
Proteomic analysis pipeline. **A**. The vitreous and retina were dissected from 16 mouse eyes. Each tissue type was pooled. **B**. Protein fractions were isolated and digested with trypsin. **C**. Peptide fragments were analyzed by multi-dimensional LC-MS/MS. **D**. MASCOT and SEAQUEST were used for peptide fragmentation finger-printing. **E**. Proteins with at least 2 peptide hits were analyzed for differential expression, ontology, pathway representation, and protein interactions.

Mouse vitreous displays features of human vitreous including zonules, vitreous base, posterior cortex and core. The vitreous from 8 mouse eyes were eviscerated as described previously[12]. Briefly, scleral tissue posterior to the limbus was grasped with a 0.22 forcep and a microsurgical blade was used to make a linear incision in the cornea from limbus to limbus. A fine curved needle holder was inserted behind the lens and then pulled forward, eviscerating both the lens and the vitreous. The lens-vitreous tissue was centrifuged with 20 microliters of protease inhibitor cocktail (Roche) dissolved in PBS. The fine curved needle holder was placed as far posterior to the globe as possible and the retina was eviscerated through the corneal incision. Some vitreous also came with the retina. The retina-vitreous tissue was also filtered. The filtered centrifuge tube was spun at 14,000 x G for 12 minutes and the eluent (vitreous) was collected (Figure 2A).

### Multidimensional protein (MudPIT) mass spectrometry

#### Protein Extraction and Digestion

Proteins were prepared for digestion using the filter-assisted sample preparation (FASP) method[13]. Briefly, the sample was added to 1% SDS, 50 mM Tris-HCl, pH 7.6, 10 mM DTT and heated at 95°C for 10 min. Samples were then transferred to a 30k Amicon MWCO device (Millipore) and centrifuged at 13 x *g* for 30 min. The remaining sample was buffer exchanged with 6 M urea, 100 mM Tris-HCl, pH 7.6, then alkylated with 55 mM iodoacetamide. Concentrations were measured using a Qubit fluorometer (Invitrogen). Trypsin was added at a 1:50 enzyme to substrate ratio and the sample was incubated overnight at 37°C. The device was centrifuged and the filtrate collected (Figure 2B).

#### Peptide Desalting and Fractionation

Digested peptides were desalted using C_18_ stop-and-go extraction (STAGE) tips[14]. Briefly, for each sample a C_18_ STAGE tip was activated with methanol, then conditioned with 60% acetonitrile, 0.5% acetic acid followed by 5% acetonitrile, 0.5% acetic acid. Samples were loaded onto the tips and desalted with 0.5% acetic acid. Peptides were eluted with 60% acetonitrile, 0.5% acetic acid and lyophilized in a SpeedVac (Thermo Savant), approximately 2 hours. Peptides were fractionated by strong anion exchange STAGE tip chromatography. Each sample was dissolved in Britton Robinson buffer pH 11 and loaded on to the STAGE tip. Flow-through was collected using a C_18_ STAGE tip. Subsequent fractions were collected by eluting peptides with Britton Robinson buffers at pH 8, 6, 5, 4 and 3.2 and capturing with C_18_ STAGE tips. Peptides were eluted from the C_18_ STAGE tip and dried as described above.

#### Liquid Chromatography-Tandem Mass Spectrometry

Each fraction was analyzed by liquid chromatography tandem mass spectrometry (LC-MS/MS) (Figure 2C). LC was performed on an Agilent 1100 Nano-flow system. Mobile phase A was 94.5% MilliQ water, 5% acetonitrile, 0.5% acetic acid. Mobile phase B was 80% acetonitrile, 19.5% MilliQ water, 0.5% acetic acid. The 120 min LC gradient ran from 5% A to 35% B over 90 min. Samples were loaded to a 2 cm x 100 um I.D. trap column positioned on an actuated valve (Rheodyne). The column was 13 cm x 100 um I.D. fused silica with a pulled tip emitter. Both trap and analytical columns were packed with 3.5 um C_18_ resin (Zorbax SB, Agilent). The LC was interfaced to a dual pressure linear ion trap mass spectrometer (LTQ Velos, Thermo Fisher) via nano-electrospray ionization. An electrospray voltage of 1.8 kV was applied to a pre-column tee. The mass spectrometer acquired tandem mass spectra from the top 15 ions in the full scan from 400 - 1400 m/z. Dynamic exclusion was set to 30 seconds. 

#### Data Processing and Library Searching

Mass spectrometer RAW data files were converted to MGF format using msconvert. All searches required strict tryptic cleavage, 0 or 1 missed cleavages, fixed modification of cysteine alkylation, variable modification of methionine oxidation and expectation value scores of 0.01 or lower. MGF files were searched using X!Hunter[15] against the latest library available in 2010 on the GPM[16] and X!!Tandem[17,18] using both the native and k-score[19] scoring algorithms and by OMSSA[20]. All searches were performed on Amazon Web Services-based cluster compute instances using the Proteome Cluster interface. XML output files were parsed and non-redundant protein sets determined. 

#### Bioinformatics

Proteins were considered identified if they had an expectation value of less than 0.01 (less then 1% chance of being a random assignment) (Figure 2D). Per 1000 proteins identified this allows about 10 false positive proteins. Once the list of proteins was curated, bioinformatic analyses were used to determine significant protein expression (Partek Geonomics Suite 6.6), gene ontology (GO terminology, Panther 7.2), represented pathways (MetaCore), and protein interactions (MetaCore) (Figure 2E).

### Statistical analysis

Using Partek Genomics Suite 6.6, the protein lists for all three mass spectrometry runs for both the vitreous and retina were analyzed. Semi-quatitative peptide hit values were set to a minimum of 0.001, setting all zero values to 0.001. Values were normalized to log base 2 and retina and vitreous results were compared using ANOVA. All statistically significant proteins (p < 0.05) were visualized using an un-discriminated clustered heatmap with a normalized clustering function. 

### Gene Ontology (GO) distribution analysis

Lists of proteins were uploaded into the Panther 7.2 Classification system under the Batch ID search menu. Pie charts were created for the visualization of GO distribution within the list of proteins. Three pie charts were created for each GO term category including biological process, molecular function, and cellular component. Pie charts were created for the individual vitreous and retina protein profiles, statistically different proteins, and common proteins.

### Pathway Analysis

MetaCore (GeneGO Inc., St. Joseph, MI, USA) OMICs data analysis was used to determine the most significant cellular pathways in the mouse vitreous and retina. MetaCore is a software program that generates protein pathway maps using curated literature databases(11). Pathways were generated by MetaCore software using Dikstra’s shortest path algorithm. The significance of the pathway is determined by the number of intersecting data points between the user’s file and a set of proteins corresponding to the particular network. More information regarding MetaCore software can be obtained at www.genego.com. The most commonly represented pathways in the vitreous and retina tissues were determined separately. Pathways were also determined for retina and vitreous differentially and commonly expressed proteins separately. 

### Network Analysis

We used the MetaCore GeneGO networking function to curate interaction maps of the proteins identified. Information for identified interactions is obtained from several sources including but not limited to genomic context, database imports (PPI and pathway databases), high-throughput experiments, co-expression, and text mining. We uploaded our lists of proteins found in the retina and the vitreous separately into the multiple sequence entry window and exported the networks into Cytoscape 2.7.0 for manipulation of the network appearance.

## Results

### Sample Collection

The retina and vitreous were eviscerated from C57BL6/J mouse eyes. To avoid large, abundant extracellular matrix proteins of the vitreous, which otherwise saturate the mass spectrometry columns and mask smaller, soluble and less abundant proteins, a 100 kDa cutoff filter was used to separate the vitreous from retina tissue by filtration and differential centrifugation. The total protein isolated from eight retinas was 380 micrograms and from eight vitreous samples was 87 micrograms. The vitreous is normally acellular. To verify the absence of retinal cellular material, vitreous samples were put onto glass slides and stained with hematoxylin and eosin. No intact cells, cell nuclei, or cell membranes were observed (data not shown). Histological analysis of lenses showed that the capsules remained intact, and there was no spillover of lens material. 

### Mass Spectrometry Overview

Samples in triplicate underwent trypsinization and multidimensional liquid chromatography before analysis by mass spectrometry. In the retina, we identified 106,735 spectra corresponding to 5,729 unique peptides, corresponding to 1,680 unique proteins (Tables S1 and S2). The vitreous was less complex showing 45,507 spectra with 1,085 unique peptides, corresponding to 675 unique proteins (Tables S1 and S2).

Rhodopsin, which is exclusively expressed by photoreceptor cells, was detected in the retina and completely absent in the vitreous, supporting the histological findings that tissues were well separated. The most abundant proteins in the retina included fifteen different crystallins, histone cluster-1, vimentin, pyruvate kinase, tubulins, ATP synthase, histone cluster-2, actin, glyceraldehyde-3-phosphate dehydrogenase, and spectrin alpha-2. The most abundant proteins in the vitreous included crystallins, fatty acid binding protein-5, diazepam binding inhibitor, phosphoglycerate kinase-1, phosphatidylethanolamine binding protein-1, ubiquitin B, triosephosphate isomerase-1, coactosin-like-1, carbonic anhydrase-2, and peroxiredoxin-5 (Table S1). 

### Differential Expression

Retinal and vitreous proteins were compared using an ANOVA 2-way statistical measurement. There were 951 retinal proteins and 44 vitreous proteins differentially expressed (p < 0.05). The remaining 729 and 631 proteins of the 1,680 and 675 total from each retina and vitreous dataset did not meet comparative statistical significance by this analysis. Several peptides were represented in both retina and vitreous (p ≥ 0.05). This group of similarly expressed peptides suggested that a number of proteins were expressed in both the retina and vitreous. 

### Gene Ontology

**Figure 3:**
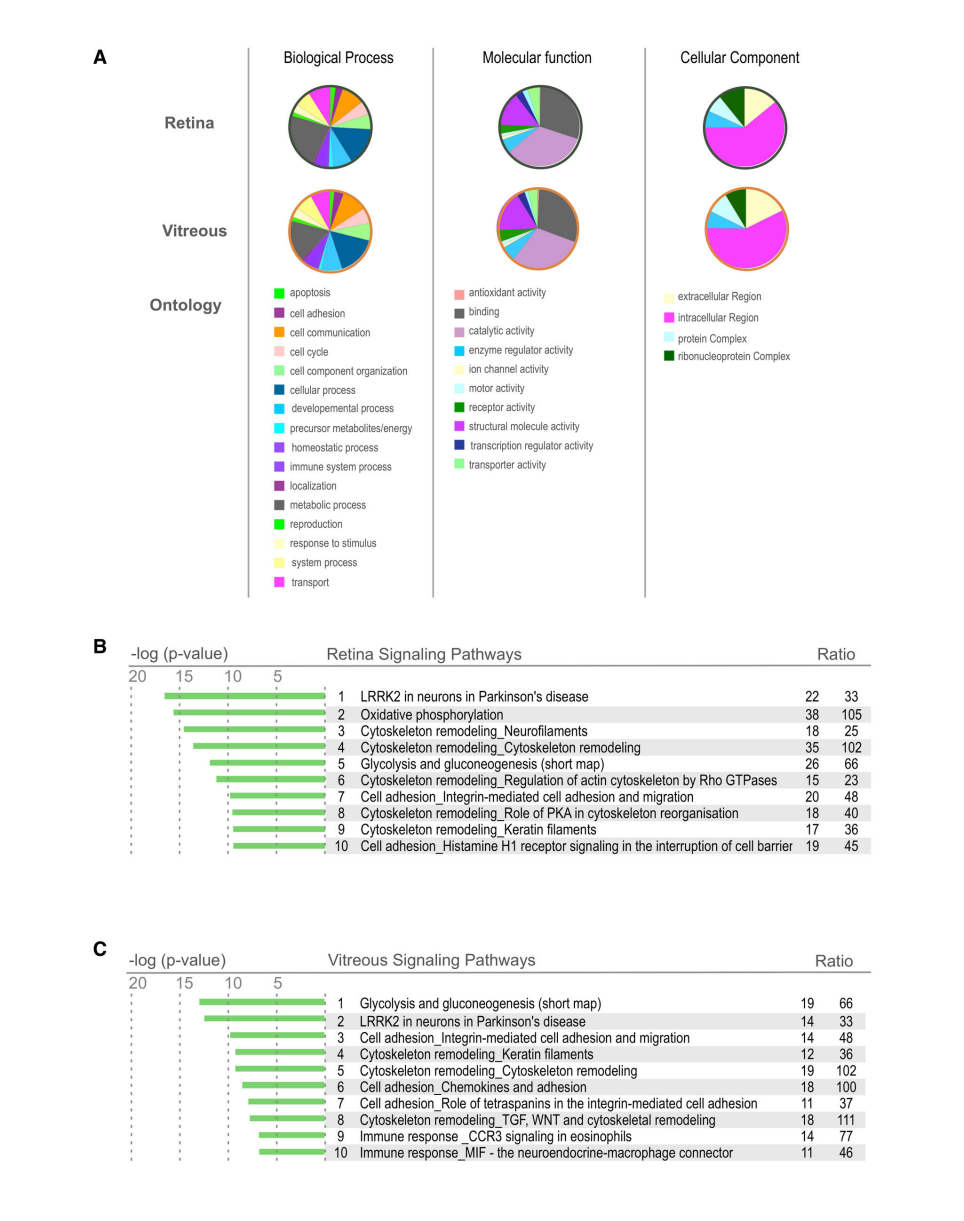
Retina and Vitreous Proteome Ontology. **A**. Heatmap showing the differentially expressed proteins of the mouse vitreous and retina. There is a large population of proteins that are overexpressed in the retina in comparison to the vitreous. Proteins that are found in this heatmap provide an excellent reference for potential disease markers. Proteins that change their expression in these tissues could be used as potential biomarkers in the future. **B**. Gene Ontology distribution of proteins differentially expressed in the mouse vitreous and retina. Proteins are grouped into sub-categories of (A.) biological processes, (B.) cellular component, and (C.) molecular processes. **C**. List of pathways most highly represented in the mouse retina and vitreous tissues. These pathways were identified using GeneGO analysis software. (statistic). .

To obtain a global view of the biological processes, molecular function, and cellular components represented by the retina and vitreous, a gene ontology analysis was performed. The retina contained higher percentages of proteins in the categories metabolic process, catalytic activity, and ribonucleoprotein complexes than the vitreous. In the vitreous there were more proteins associated with immune system process, antioxidant activity, and extracellular regions compared to the retina. (Figure 3A). The vitreous is an acellular extracellular matrix, however, intracellular proteins were highly represented in the vitreous. These included intracellular signaling and cytoskeletal molecules that are typically not expected to have a function in the extracellular environment (such as proteins involved in glycolysis). This suggests that the retina may be releasing a number of intracellular proteins into the vitreous, either as degraded byproducts or functional proteins. 

**Figure 4:**
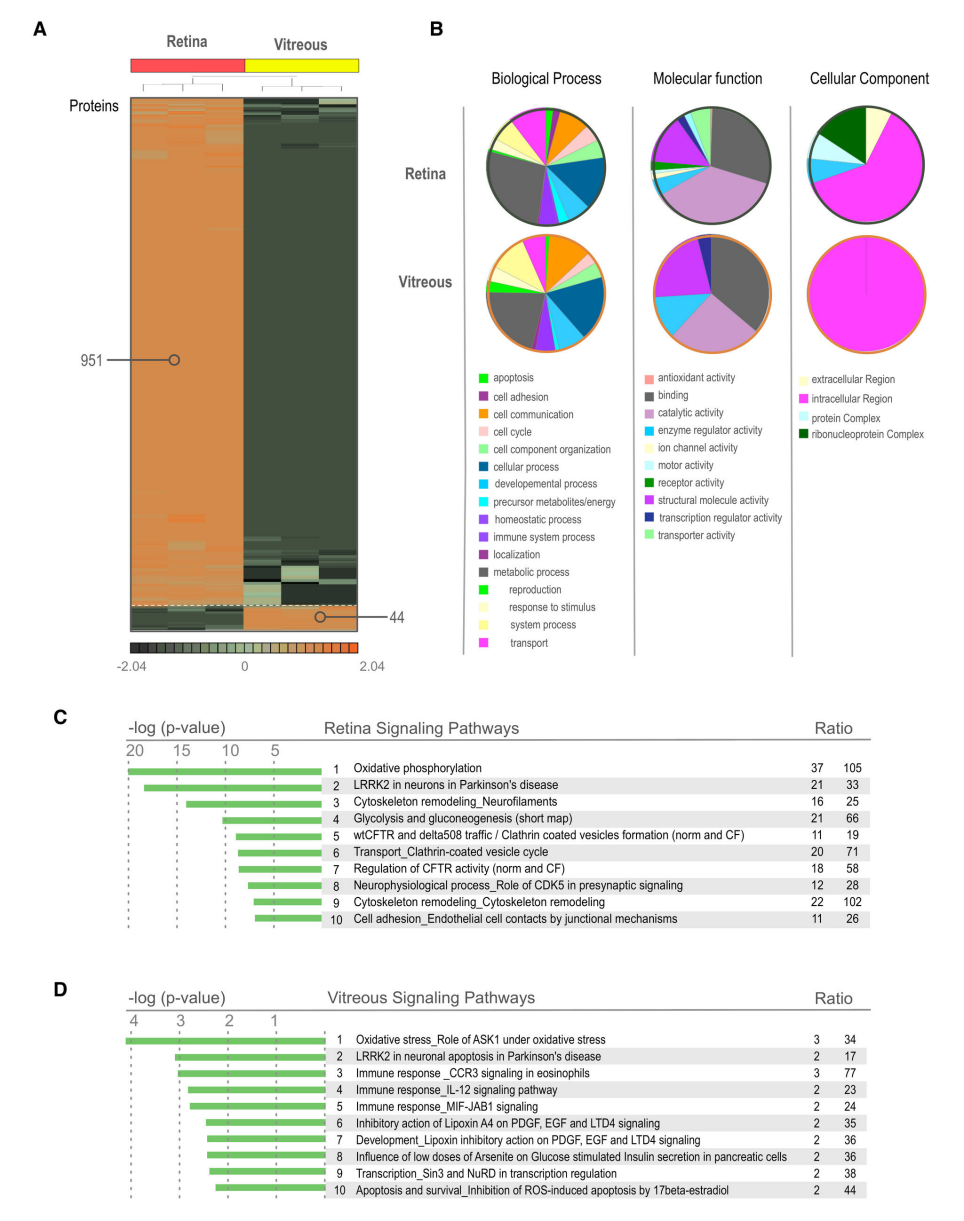
Differentially expressed proteins in the Retina and Vitreous. **A**. Heatmap showing the differentially expressed proteins of the mouse vitreous and retina (p < 0.05). There is a large population of proteins that are overexpressed in the retina in comparison to the vitreous. Proteins that are found in this heatmap provide an excellent reference for potential disease markers. Proteins that change their expression in these tissues could be used as potential biomarkers in the future. **B**. Gene Ontology distribution of proteins differentially expressed in the mouse vitreous and retina. Proteins are grouped into sub-categories of (A.) biological processes, (B.) cellular component, and (C.) molecular processes. **C**. List of pathways most highly represented in the mouse retina and vitreous tissues. These pathways were identified using GeneGO analysis software.

Differentially expressed proteins had more variability in gene ontology (GO) categorization. Proteins associated with antioxidant activity, protein complexes, and ribonuceoprotein complexes were almost exclusively associated with the retina, and proteins associated with enzyme regulator activity, structural molecule activity, and intracellular regions were more highly associated with the vitreous (Figure 4A, B). Since the vitreous does not synthesize proteins, many of the differentially expressed intracellular proteins are likely to originate in nearby cellular tissues. These include the retina, ciliary body, iris, and serum.

**Figure 5:**
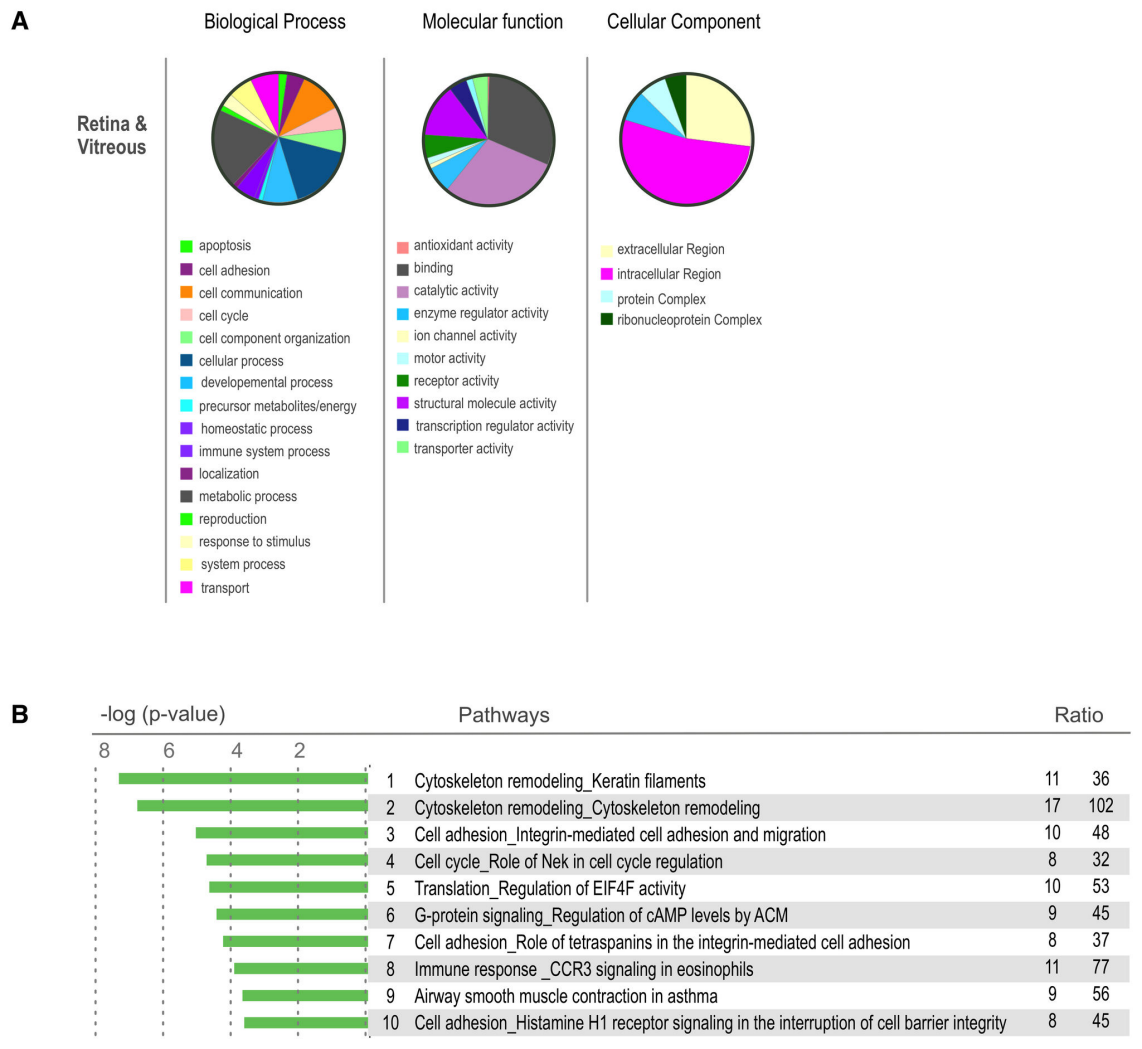
Proteins expressed similarly in the Retina and Vitreous. **A**. Gene Ontology distribution of proteins identified in both the mouse vitreous and retina. Proteins are grouped into sub-categories of (A.) biological processes, (B.) cellular component, and (C.) molecular processes. **B**. Top 50 pathways represented by the similar proteins (p >= 0.05).

The proteins detected in both the retina and the vitreous were also analyzed. Interestingly, intracellular proteins was the largest group of proteins represented for cellular component (Figure 5A). Metabolic process was the largest represented group for biological processes and catalytic activity was the largest molecular function group. Thus, the gene ontology analysis supported the concept that the vitreous is comprised of many proteins secreted by the retina or originally from the retina, possibly as break down products.

### Molecular Pathways

A molecular pathway analysis identified groups of functionally related proteins in the retina and vitreous. Pathways were ranked based on a ratio of the number of proteins identified in our samples to the total number if proteins in the pathway as determined by MetaCore. The top ten pathways with the highest representation in the retina were LRRK2 in neurons, oxidative phosphorylation, 5 classes of cytoskeleton remodeling, glycolysis and gluconeogenesis, cell adhesion for integrin-mediated adhesion, and migration as well as for histamine H1 receptor signaling (Figure 3B/Table S3). 

Since pathway databases are manually curated with overlapping molecular relationships, pathway names did not always clearly denote the key molecular components. In the LRRK2 pathway, for example there was no LRRK2, a protein associated with Parkinson’s disease. Instead this pathway was represented by a number of related markers for neuronal cells, including proteins associated with presynaptic vesicles and actin cytoskeletal reorganization. The retina is the most metabolically active tissue in the body, and not surprisingly, mitochondrial proteins involved in oxidative phosphorylation formed the second most highly represented pathway. Overall, the major molecular pathways were consistent with a neural tissue of high energy consumption and significant G-protein signaling.

The top ten pathways found in the vitreous included glycolysis and gluconeogenesis, LRRK2 in neurons in Parkinson’s disease, three classes of cell adhesion, three classes of cytoskeletal remodeling, CCR3 mediated immune response, and MIF mediated immune response (Figure 3C/Table S4). Some of the pathways were very similar to those found in the retina, indicating, again, that retinal proteins were also represented in the vitreous. These include LRRK2, cell adhesion, and cytoskeleton remodeling.

The most highly represented pathways that distinguished the retina from the vitreous (p < 0.05) were oxidative phosphorylation, LRRK2 in neurons, 2 classes of cytoskeleton remodeling, glycolysis and gluconeogenesis, wtCFTR and delta508 trafficking, clathrin coated vesicle transport, regulation of CFTR activity, neurophysical processes, and cell adhesion for endothelial cell contacts (Figure 4C/Table S5). Pathways that distinguished the vitreous from the retina (p < 0.05) were oxidative stress, immune responses mediated by CCR3, IL-12, MIF-JAB1, inhibitory action of lipoxin A_4_ on PDGF, EGF and LTD4, influence of arsenite on glucose, Sin3 and NuRD mediated transcription, and ROS-induced apoptosis/survival (Figure 4D/Table S6).

Interestingly, there were instances in which both the retina and vitreous expressed different sets of proteins that were part of the same pathway. For example, different components of LRRK2 in neurons were each present in the vitreous and retina. Other pathways that were common to both the retina and vitreous were cytoskeleton remodeling, cell adhesion, cell cycle, translation, G-protein signaling, immune response mediated by CCR3, and smooth muscle contraction (Figure 5B/Table S7). The detection of multiple cell adhesion and cytoskeleton pathways fit with the expected extracellular matrix protein composition of the vitreoretinal interface. The finding of numerous intracellular proteins not expected to be functional in the vitreous was not expected.

### Interacting proteins in the retina and vitreous identified using network analysis

**Figure 6:**
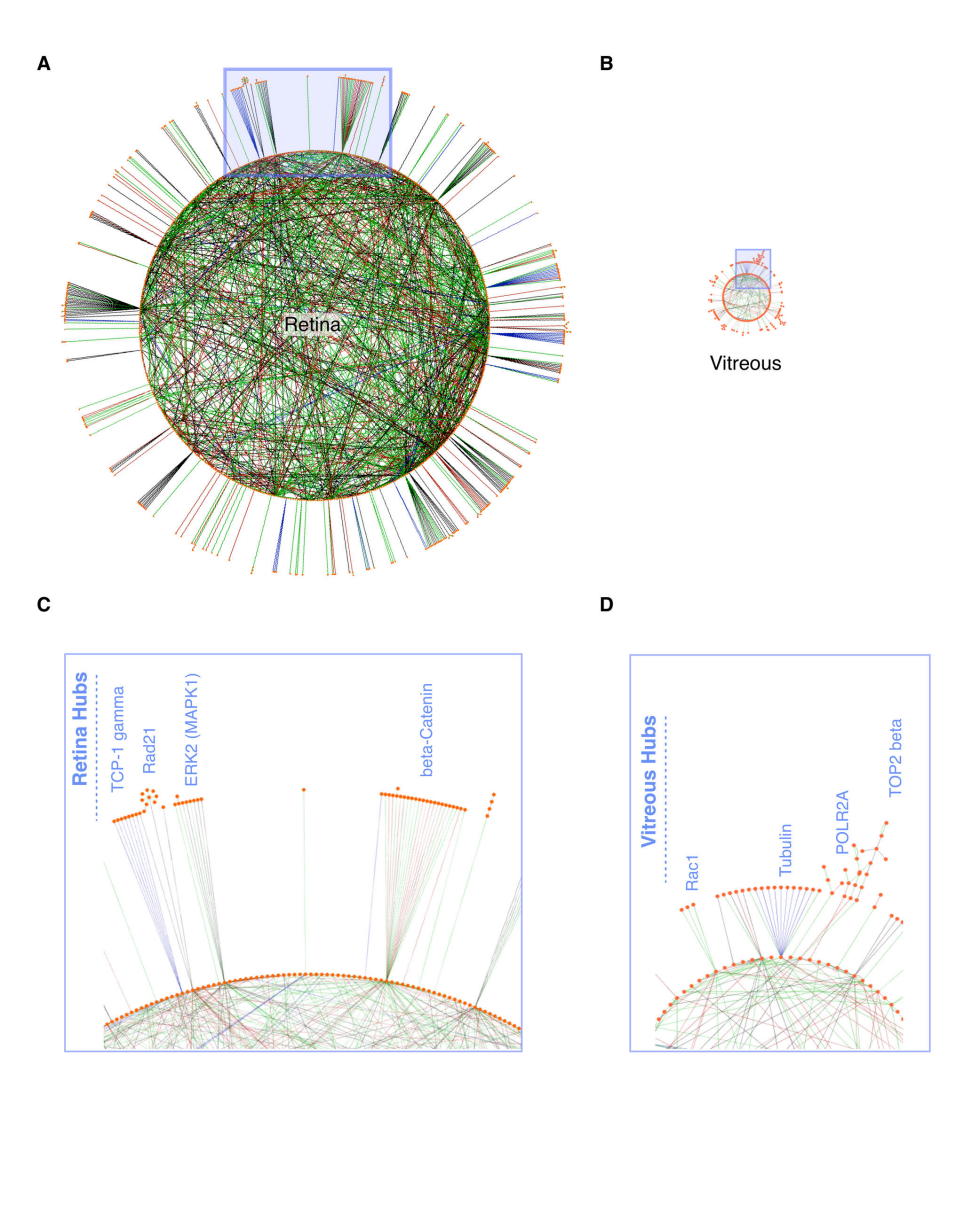
Mouse retina and vitreous protein networks. **A**-**B**. Circular layout of the mouse retina and vitreous networks. Nodes (individual proteins; orange filled circles) with no edges (interactions) were not visualized. Nodes in the vitreous and retina are sized to the same scale to emphasize the difference in complexities of the two tissues. Enlarged blue box regions showing examples of hubs from the retina (**C**) and vitreous (**D**). Edges denote interactions (binding, substrate, transcriptional target) and are color coded (green = activation; red = inhibition; black = unknown).

**Figure 7:**
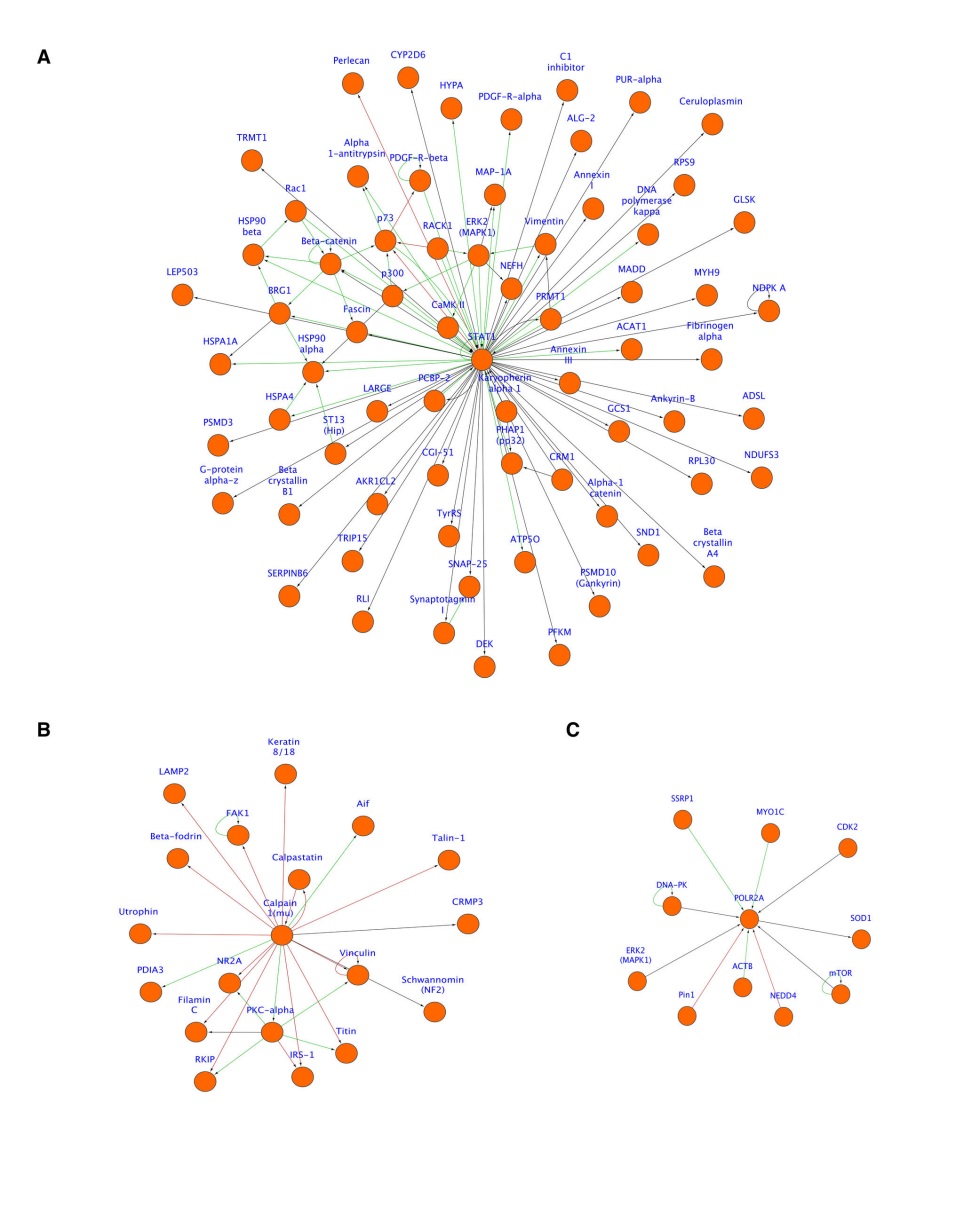
Mouse vitreoretinal protein hubs associated with human disease. **A**. Retinal STAT1 hub showing proteins associated with STAT1 pathways in the eye through specific interactions. STAT1 is a transcription activator associated with disease. **B**. Calpain-1 hub expanding pathways associated with calpain-1 in the mouse vitreous. Calpain-1 is an intracellular cysteine protease. **C**. Superoxide dismutase 1 (SOD1) and associated proteins were also found in the mouse vitreous. SOD1 is elevated in the vitreous of diabetic patients. Edges denote interactions (binding, substrate, transcriptional target) and are color-coded (green = activation; red = inhibition; black = unknown).

Direct physical interaction networks were assembled from whole retina and vitreous proteomes (Figure 6A, B). The retina had 1616 nodes, 1668 edges, and 168 hubs (Figure 6A). The number of nodes was fewer than the input protein list (1682) because not every protein was recognized by MetaCore. The vitreous was less complex with only 624 nodes, 311 edges, and 28 hubs (Figure 6B). As shown, the retina had many distinct hubs, some of which had multiple interacting proteins on the circle and outside the circle (Figure 6A, C). Outside hubs indicate proteins that interact with a single protein in the tissue network but also interact with another unique set of proteins. The vitreous hubs were less complex than those seen in the retina (Figure 6B, D). The relative size of each network was also informative. The retina network was larger than that of the vitreous, indicating the larger number of interacting proteins (nodes) as well as the higher degree of complexity (higher numbers of edges and hubs). Viewing single nodes in more detail helped determine all of the interacting proteins for one protein. For example, there are several interacting proteins for STAT1 in the retina including p73, p300, Annexin, perlecan, and beta-catenin (Figure 7A). We also found an interaction network for the intracellular protein calpain-1 in the vitreous, including calpastatin, talin-1, aif, and LAMP2 (Figure 7B). Another protein hub we found in the vitreous was SOD1 (Figure 7C). This is an intracellular protein involved in oxidative stress. Interacting proteins include MYO1C, CDK2, POLR2A, mTOR NEDD4, ACTB, Pin1, and SSRP1. The finding of entire physical interaction hubs suggest these protein complexes were released as a group that could be functional or degraded byproducts.

### Human vitreoretinopathy biomarker validation

The mouse dataset was interrogated to determine if there was any correlation to previously reported proteins that change expression levels in human vitreoretinal disease.

There are several soluble biomarkers reported for age-related macular degeneration (AMD), proliferative diabetic retinopathy (PDR), proliferative vitreoretinopathy (PVR), retinal detachment (RD), and uveitis. Some of these biomarkers include catalase (Cat), peroxiredoxins (Prdx), crystallins (Cry), glutathione peroxidase (Gpx), S100, complement proteins, vascular endothelial growth factor (VEGF), carbonic anhydrase, and macrophage migration inhibitory factor (Mif)[21-26]. Each of these were found in our mouse dataset (Table 1). Many of the biomarkers are found in more than one disease. For example, VEGF is a biomarker found in AMD, PDR, and PVR[22,27,28].

**Table 1:** Biomarkers of human vitreoretinal diseases.

**Human Biomarker Symbol**	**Description**	**Average Mouse Retina Peptide Hits**	**Average Mouse Vitreous Peptide Hits**
C3	Complement C3	1	0
Capn1	Calpain 1	1	0
Capns1	Calpain small subunit 1	8	0
Capn2	Calpain 2	2	0
Car2	Carbonic anhydrase II	80	90
Car3	Carbonic anhydrase III	1	21
Cat	Catalase	10	1
Cryaa	Crystallins	10,701	682
Got1	Glutamate oxaloacetate transaminase	121	10
Gpx4	Glutathione peroxidase, nuclear	10	6
GST1	Glutathione-S-Transferase	9	4
Mif	Macrophage migration inhibitory factor	116	86
Nos2	Nitric oxide synthase 2	0	5
PDGFr	Platelet derived growth factor receptor (alpha and beta)	10	0
PEDF	Pigment epithelium-derived factor	0	0
Prdx1	Peroxiredoxin 1	100	8
Prdx2	Peroxiredoxin 2	122	14
Prdx3	Peroxiredoxin 3	14	0
Prdx4	Peroxiredoxin 4	38	2
Prdx5	Peroxiredoxin 5	123	87
Prdx6	Peroxiredoxin 6	76	32
S100	S100 precursors A6 and A11	39	21
Sod1	Superoxide dismutase [Cu-Zn]	2	2
VEGF	Vascular endothelial growth factor	0	0
vWF	Von willebrand factor precursor	19	0

Peptide hits are listed for proteins found in mouse vitreous and/or retina.

Some biomarkers are absent or very low in the normal vitreous, but elevated in human disease. We found these were also absent from mouse vitreous. These included Sod1, Nos2 (low), VEGF, PEDF, PDGFr, Prdx3, vWF, and C3 (Table 1). VEGF and PEDF were absent in both the normal human vitreous and mouse. In human ischemic retinopathies, these proteins drive pathogenic angiogenesis, and they are potential biomarkers in mouse models of retinal angiogenesis[29]. Taken together, the mouse dataset correlates with several known proteins associated with human vitreoretinal disease. This supports the potential of modeling specific vitreoretinal diseases in the mouse and exploiting the same biomarkers.

## Discussion

Proteomic analysis is especially important in the examination of complex extracellular matrices such as the vitreous where both ocular and remote tissues contribute to the diseased state. In these cases, analysis of local tissue gene expression may provide a very limited view of disease pathophysiology. The purpose of our study was to create a reference dataset of the proteomic interactions between the mouse vitreous and retina. Mass spectrometry identification of peptides can be more sensitive and specific than Western blot techniques. It allows simultaneous identification of hundreds of proteins without the variability associated with antibodies. Overall, our study supports the use of the mouse as model for human vitreoretinal disease. It also reveals novel functions of the vitreous and provides insight into nonstructural interactions with the retina.

Proteins identified in the mouse vitreous included both extracellular and intracellular proteins. Extracellular and cytoskeletal proteins were expected as the vitreous is a large extracellular matrix. Also, immune response proteins were expected because they have been identified in other reports and our own studies of the human vitreous. We also found that the proteins in the vitreous contain a subset of proteins that are specific to the retina. Proteins from the retina may populate the vitreous normally as ‘shed’ byproducts of retinal cell turnover. This idea is supported by previous reports of retinal proteins found in the vitreous of humans[24,26]. Alternatively, some of these proteins may function normally in the vitreous. In either case, the vitreous may serve as an indirect source to biopsy the retina.

Other tissues in contact with the vitreous include the ciliary body, iris, and lens, and all of these structures are potential sources of vitreous proteins. Proteins circulating in the blood, such as albumin and IgG, were found in the vitreous. Intracellular proteins identified in the vitreous that should only be present in the retina may comprise the retinal ‘degradome’, proteins that are broken down and spilled into the vitreous as a waste removal mechanism. These degraded proteins provide great insight into retinal turnover in health and disease. We also found several crystallins in the vitreous and retina. Crystallins are chaperone proteins expressed in many tissues[30,31], and were present in both the retina and vitreous. The top 20 retina proteins contained fifteen different crystallins, which are upregulated in the retina during the progression of ocular diseases, including uveitis[30,32,33]. Thus, multiple cells and tissues, including those outside the eye, may contribute to the vitreous proteome.

Vitreous proteins are emerging as effective targets in ocular disease therapy. VEGF is inhibited by anti-VEGF antibodies in age-related macular degeneration and diabetic retinopathy. Proteolysis of laminin, fibronectin and type 4 collagen by ocriplasmin leads to posterior vitreous detachment and macular hole closure[34,35]. Comprehensive analysis of the mouse retina and vitreous body may lead to a greater understanding of human vitreoretinal diseases and the discovery of potential therapeutic targets. 

Proteins found in the vitreous of human disease, (i.e. C3, SOD1), and not found in the control mouse vitreous offer good biomarker potential for evaluating mouse models of disease. Other biomarkers that were found in the control mouse vitreous (i.e. Mif) can be used as a baseline for comparison. Mouse models of AMD, for example, include the *Sod1-/-* (and *Sod1+/-*) and the *Ccl2-/-* (and *Cclr-/-*) mice, which exhibit altered expression of proteins including Sod1, VEGF, and C3[36-38]. Vitreous biopsies are used in the diagnosis of intraocular infections, but diagnostic and therapeutic delays occur due to the time required for organism culture and the poor sample quality for PCR. Proteomic analysis of inflammatory vitreous fluid may identify biomarkers that can be assayed much faster, and mouse models of endophthalmitis can be used to test this hypothesis.

This study provides insight into the normal composition of retinal and vitreous proteins as a reference for other mouse models of disease. Intracellular proteins that were found in the vitreous of the mouse that should be found in the retina indicate potential byproducts of the retina. Analyzing these protein profiles in healthy individuals compared to diseased individuals will give insight into biomarkers and therapeutic targets. Our mouse vitreoretinal proteome provides a baseline for discovering these unique proteins in mouse models of human ocular diseases.

## Supporting Information

Table S1 **Complete table of proteins identified in the mouse vitreous and retina using LC-MS/MS.** Proteins in the table are identified by their Ensembl ID, gene ID, description, the number of unique peptides, the number of peptide hits, and identification of equivalent proteins.
(XLSX)

Table S2 **Peptides identified for proteins found in the mouse vitreous and retina listed in Table S1.**
(XLSX)

Table S3 **Top 50 differentially expressed pathways in the retina.**
(XLS)

Table S4 **Top 50 differentially expressed pathways in the vitreous.**
(XLS)

Table S5 **Top 50 differentially expressed pathways representing proteins that are statistically different in the retina compared to the vitreous.**
(XLS)

Table S6 **Top 50 differentially expressed pathways representing proteins that are statistically different in the vitreous compared to the retina.**
(XLS)

Table S7 **Top 50 differentially expressed pathways representing proteins that are statistically similar between the retina and the vitreous.**
(XLS)
